# Finite element analysis of biological soft tissue surrounded by a deformable membrane that controls transmembrane flow

**DOI:** 10.1186/s12976-018-0094-9

**Published:** 2018-12-10

**Authors:** Satoko Hirabayashi, Masami Iwamoto

**Affiliations:** Toyota Central R & D Labs., Inc., 41-1, Yokomichi, Nagakute, Aichi, Japan

**Keywords:** Finite element method, Large deformation, Pore water pressure, Mixture theory, Membrane, Hydrated poroelastic material

## Abstract

**Background:**

Many biological soft tissues are hydrated porous hyperelastic materials, which consist of a complex solid skeleton with fine voids and fluid filling these voids. Mechanical interactions between the solid and the fluid in hydrated porous tissues have been analyzed by finite element methods (FEMs) in which the mixture theory was introduced in various ways. Although most of the tissues are surrounded by deformable membranes that control transmembrane flows, the boundaries of the tissues have been treated as rigid and/or freely permeable in these studies. The purpose of this study was to develop a method for the analysis of hydrated porous hyperelastic tissues surrounded by deformable membranes that control transmembrane flows.

**Results:**

For this, we developed a new nonlinear finite element formulation of the mixture theory, where the nodal unknowns were the pore water pressure and solid displacement. This method allows the control of the fluid flow rate across the membrane using Neumann boundary condition. Using the method, we conducted a compression test of the hydrated porous hyperelastic tissue, which was surrounded by a flaccid impermeable membrane, and a part of the top surface of this tissue was pushed by a platen. The simulation results showed a stress relaxation phenomenon, resulting from the interaction between the elastic deformation of the tissue, pore water pressure gradient, and the movement of fluid. The results also showed that the fluid trapped by the impermeable membrane led to the swelling of the tissue around the platen.

**Conclusions:**

These facts suggest that our new method can be effectively used for the analysis of a large deformation of hydrated porous hyperelastic material surrounded by a deformable membrane that controls transmembrane flow, and further investigations may allow more realistic analyses of the biological soft tissues, such as brain edema, brain trauma, the flow of blood and lymph in capillaries and pitting edema.

## Background

**Fig. 1:**
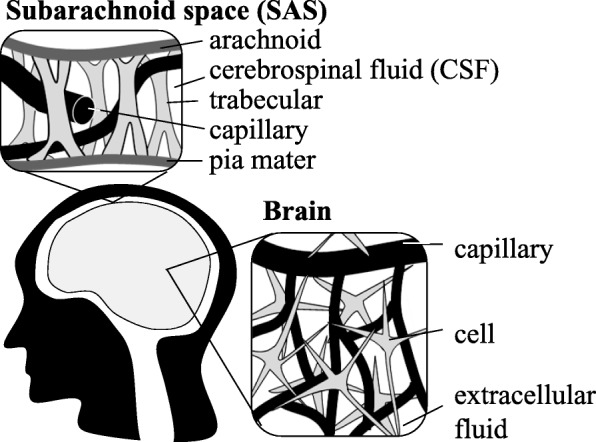
Diagrams of hydrated soft tissues in head. The subarachnoid space (SAS) is located between the arachnoid and the pia mater, and it contains a trabecular network, capillaries, and cerebrospinal fluid (CSF) The arachnoid is generally parallel to the pia mater, which adheres to the brain surface, except for the covering of brain sulci. Brain contains with a cellular network, capillaries, and extracellular fluid

Many body parts represent hydrated soft tissues that resembles a water filled sponge, consisting of the complex solid skeleton with multiple fine voids and fluid that fills the voids. Capillaries form a network of blood-filled vessels throughout the body, while cells contain various organelles and intracellular fluid filling the remaining space and the extracellular space is filled with extracellular fluid. In the head, the subarachnoid space (SAS) between the skull and the brain is filled with a trabecular network and cerebrospinal fluid (CSF) [1–5] (Fig. 1). Most of these tissues are surrounded and partitioned by membranes, and transmembrane flows are controlled by various mechanisms such as valves, barriers, and channels. Solids and fluids interact mechanically, especially through the pore pressures of fluids confined by membranes. The SAS collapse after CSF draining [5] suggests that the pore pressure of the CSF confined in the SAS stabilizes a weak skeleton. SAS thickness can change with the CSF flow along the membrane, which allows the brain to approach the skull. Here, the resistance of the trabeculae and membranes slow down the CSF flow, reducing brain acceleration and protecting the brain. While pore pressures protect the brain in SAS, they might harm it as well. Transmembrane fluid flow disorders can cause cerebral edema, defined as an increase in the brain internal fluid contents: intracellular fluid (cytotoxic edema), interstitial fluid (vasogenic edema), and CSF (interstitial edema or hydrocephalic edema). Consistent with the Monroe-Kellie doctrine, cerebral edemas lead to the development of severe conditions, such as cerebral ischemia, intracranial pressure (ICP) elevation, and intracranial compartmental shifts, resulting in the compression of vital brain structures (herniation) [6].

Finite element method (FEM) is an effective tool for the investigation and prediction of these phenomena. However, the complex micro-level solid-fluid boundaries in the hydrated soft tissues require microscopically fine meshes, making FEM analysis unrealistic. Therefore, FEMs based on the classical consolidation theory (or more rigorous and versatile mixture theory) have been developed. According to the biphasic theory, which is a mixture theory considering a two-phase mixture of solid and fluid phases, the movement of each microscopic component is treated separately. By considering the averaged interactions between them, the mixture is then macroscopically analyzed. Since hydrated biological tissues generally experience large deformations, Oomens et al. [7] developed a nonlinear mixed finite element formulation of mixture theory by using pressure and solid displacement as the nodal unknowns. To assume the solid phase to be hyperelastic, Suh et al. [8] developed a new nonlinear penalty finite element formulation by using solid displacement and fluid velocity as the nodal unknowns. Levenston et al. [9] described two newer three-field (solid displacement, fluid velocity, and pressure) mixed finite element formulations and demonstrated the improved performances of the formulations over an analogous two-field (solid displacement and fluid velocity) penalty formulation. These FEMs have been developed to treat more complicated phenomena [10–13] involving multi-physics phenomena such as charged hydrated tissues [14–19]. In these studies, most of the boundaries of the tissues were treated as rigid and/or freely permeable. Despite the importance of the transmembrane flow control by deformable membrane we mentioned above, these studies didn’t analyze the tissues with the boundaries of deformable membranes that control the transmembrane flows.

**Fig. 2:**
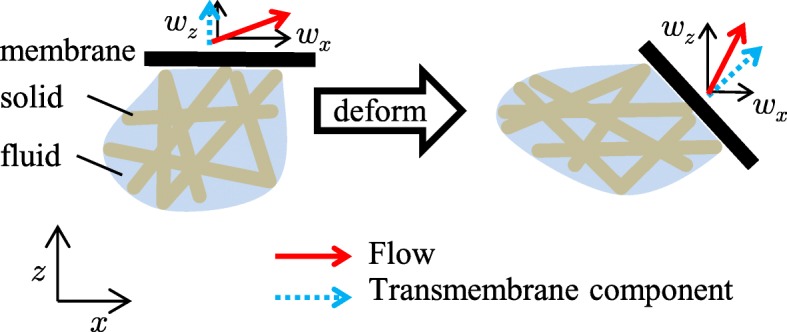
The flow components under the deformation. Red bold solid lines represent the flows at the membrane. Blue bold dashed lines represent the transmembrane flow, a flow velocity component perpendicular to the membrane. By setting the *z*-axis along the normal vector of the membrane, the transmembrane flow coincides with the flow velocity *z*-component *w*_*z*_ in the initial states. However, when a large deformation occurs, the normal vector of the membrane does not coincide with one of the axes *x*, *y* (not illustrated) or *z*, and the transmembrane flow does not coincide with the axis component of the flow velocity *w*_*x*_, *w*_*y*_ (not illustrated) or *w*_*z*_

The aim of the present study was to develop an FEM for the analysis of hydrated biological soft tissues surrounded by deformable membranes which control the transmembrane flows. The transmembrane flow is regarded as the flow velocity component perpendicular to the membrane (Fig. 2). In the FEMs formulated by Suh et al. [8] and Levenston et al. [9], the fluid velocity vector at each node is divided into a set of three scalar nodal unknowns, that is, a set of axis components. By setting any axis perpendicular to the membrane, the axis component coincides with the transmembrane flow, which is the flow velocity component perpendicular to the membrane (Fig. 2), and can be provided as the Dirichlet boundary condition. However, the deformable membrane generally rotates during large deformations while the axes are fixed. Thus, these FEMs might not be applied to deformable membranes. Therefore, in this study, we developed a new nonlinear FEM based on the mixture theory by using pressure and solid displacement as the nodal unknowns and controlling the transmembrane flow volume using Neumann boundary condition.

## Methods

### Preliminaries of continuum mixture theory

**Fig. 3:**
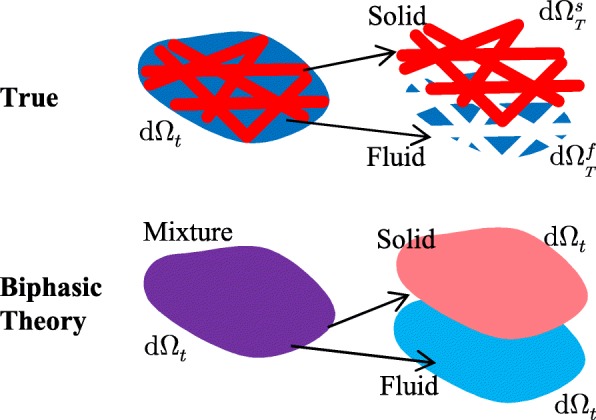
True and apparent configurations of a solid-fluid mixed material according to the biphasic theory. In the true configuration, the space d*Ω*_*t*_ can be divided in the solid space $\mathrm {d}\Omega _{T}^{s}$ and the fluid space $\mathrm {d}\Omega _{T}^{f}$. In the biphasic theory, these discontinuous spaces are assumed to be macroscopically continuous and to occupy the whole mixture space d*Ω*_*t*_. Therefore, two constituents occupy the same physical space at the same time

Although the finite element formulations of the mixture theory have been developed to be applicable for multi-physics phenomena, such as charged hydrated tissues [14–19], we focused on the biphasic theory [7–13]. Within the framework of the biphasic theory, a mixture can be treated as one continuum. Two constituents of the mixture are assumed to be macroscopically continuous and to occupy the whole mixture space. Therefore, they occupy the same physical space at the same time (Fig. 3).

In the absence of body and inertial forces, the momentum equation of the mixture is

1 $$ \boldsymbol{\nabla}_x \cdot \boldsymbol{\sigma} = \boldsymbol{0}   $$

where ***∇***_*x*_ represents the gradient operator with respect to the current configuration and ***σ*** is the Cauchy stress tensor for the mixture (total stress). For a fully saturated mixture with incompressible constituents, the Cauchy stress is

2 $$ \boldsymbol{\sigma}= -p \boldsymbol{I} + \boldsymbol{\sigma}^{E}  $$

where *p* is the fluid (pore) pressure, ***I*** is the rank-two identity tensor, and ***σ***^*E*^ is the stress induced by solid deformation [7–10]. The stress ***σ***^*E*^ corresponds to the so-called effective stress described in the classical consolidation theory [7, 10, 20].

**Fig. 4:**
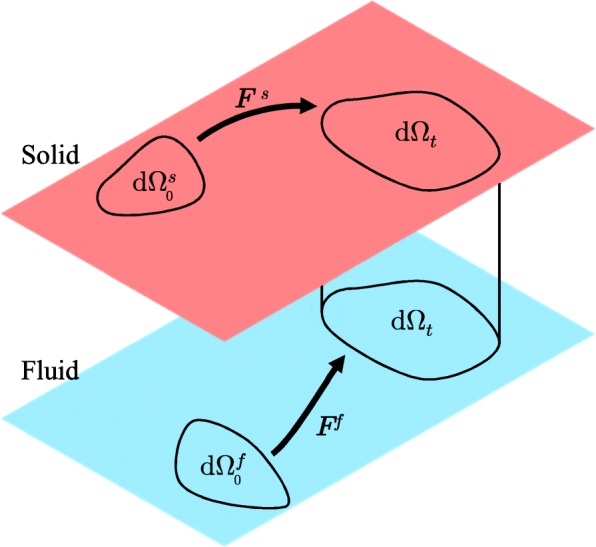
The kinematics of two phases constituting a mixed material. In the solid phase, the current micro-volume d*Ω*_*t*_ is related to the original volume $\mathrm {d}\Omega _{0}^{s}$ by the solid deformation gradient tensor ***F***^*s*^. In the fluid phase, it is associated with the original volume $\mathrm {d}\Omega _{0}^{f}$ by the fluid deformation gradient tensor ***F***^*f*^. Generally, $\mathrm {d}\Omega _{0}^{s}$ and $\mathrm {d}\Omega _{0}^{f}$ don’t coincide

In the following equations, *α*∈{*s*,*f*} denotes a quantity related to a particular constituent *α* (*s*: solid and *f*: fluid). The original micro-volume, $\mathrm {d}\Omega _{0}^{\alpha }$, is related to the current volume d*Ω*_*t*_ as:

3 $$ \mathrm{d}\Omega_{t} = J^{\alpha} \mathrm{d}\Omega_{0}^{\alpha},   $$

where the Jacobian *J*^*α*^ is the determinant of the deformation gradient tensor, ***F***^*α*^ (Fig. 4). They are defined as

4 $$\begin{array}{*{20}l}\mathrm{d}\boldsymbol{x}^{\alpha} =\boldsymbol{F}^{\alpha} \cdot \mathrm{d}\boldsymbol{X}^{\alpha} \end{array} $$

5 $$\begin{array}{*{20}l} J^{\alpha} \equiv \text{det}\boldsymbol{F}^{\alpha}, \end{array} $$

when the original infinitesimal line segment d***X***^*α*^ deforms to d***x***^*α*^ in the current configuration. Since *α* is intrinsically incompressible, the true volume of *α* is constant during the deformation. Then, the current and original volume fractions *ϕ*^*α*^ and $\phi ^{\alpha }_{0}$ can be defined as:

6 $$ \phi^{\alpha} = \frac{\mathrm{d}\Omega_{T}^{\alpha}}{\mathrm{d}\Omega_{t}}, \hspace{20pt} \phi^{\alpha}_{0} = \frac{\mathrm{d}\Omega_{T}^{\alpha}}{\mathrm{d}\Omega_{0}^{\alpha}},  $$

where $\mathrm {d}\Omega _{T}^{\alpha }$ is the true volume of *α* in d*Ω*_*t*_ of the mixture (Fig. 3). By using the Eq. (3), *ϕ*^*α*^ can be related to the initial volume fraction $\phi ^{\alpha }_{0}$ as follows:

7 $$ \phi^{\alpha}= \frac{\phi^{\alpha}_{0}}{J^{\alpha}}.   $$

### Incompressible condition

In the following equations, the material and spatial time derivative of an arbitrary quantity $\mathcal {A}$ are represented by $\dot {\mathcal {A}}$ and $\partial \mathcal {A}/ \partial t$, respectively. Then, the incompressible condition of *α* can be represented as:

8 $$ \dot{\rho}_{T}^{\alpha} = 0,   $$

where $\rho _{T}^{\alpha }$ is the true density of *α*. The *α*-velocity field ***v***^*α*^ is defined as:

9 $$ \boldsymbol{v}^{\alpha} \equiv \dot{\boldsymbol{x}}^{\alpha},  $$

where ***x***^*α*^ is the position vector of *α*.

The total mass of *α* in the volume space *Ω*_*t*_, denoted by *m*^*α*^, can be written as:

10 $$ m^{\alpha} = \int_{\Omega_{t}} \rho_{T}^{\alpha} \phi^{\alpha} \mathrm{d}\Omega_{t} = \int_{\Omega_{0}^{\alpha}} \rho_{T}^{\alpha} \phi^{\alpha} J^{\alpha} \mathrm{d}\Omega_{0}^{\alpha},  $$

where $\Omega _{0}^{\alpha }$ denotes the original volume space of *α* occupying *Ω*_*t*_ at current time. Therefore, using Eq. (8) and the well-known relationship $\dot {J}^{\alpha } = J^{\alpha } \boldsymbol {\nabla }_{x} \cdot \boldsymbol {v}^{\alpha }$, the principle of the conservation of mass can be rewritten as:

11 $$ \dot{m}^{\alpha} = \rho_{T}^{\alpha} \int_{\Omega_{0}^{\alpha}} \left(\dot{\phi^{\alpha}} J^{\alpha} + \phi^{\alpha} J^{\alpha} \boldsymbol{\nabla}_{x} \cdot\boldsymbol{v}^{\alpha} \right)\mathrm{d}\Omega_{0}^{\alpha} = \rho_{T}^{\alpha} \int_{\Omega_{t}} \left(\dot{\phi^{\alpha}} + \phi^{\alpha} \boldsymbol{\nabla}_{x} \cdot\boldsymbol{v}^{\alpha} \right)\mathrm{d}\Omega_{t} = 0.  $$

As this equation holds for any arbitrary parts, we obtained the relationship

12 $$ \dot{\phi^{\alpha}} + \phi^{\alpha} \boldsymbol{\nabla}_{x} \cdot\boldsymbol{v}^{\alpha} = 0  $$

that can be rewritten as:

13 $$ \frac{\partial\phi^{\alpha}(x,t)}{\partial{t}} + \boldsymbol{\nabla}_{x} \cdot (\phi^{\alpha}\boldsymbol{v}^{\alpha}) = 0.   $$

The total sum of this equation related to the solid and the one related to fluid phases is [8, 9]

14 $$ \boldsymbol{\nabla}_{x} \cdot (\phi^{s} \boldsymbol{v}^{s} +\phi^{f} \boldsymbol{v}^{f}) = 0   $$

since

15 $$ \phi^{s}(\boldsymbol{x}, t) + \phi^{f}(\boldsymbol{x}, t) =1   $$

for a fully saturated mixture. This equation can be rewritten again [7, 10]:

16 $$ \boldsymbol{\nabla}_{x}\cdot\left(\boldsymbol{v}^{s}+\boldsymbol{w}\right)= 0,   $$

where the relative fluid velocity ***w*** is defined as:

17 $$ \boldsymbol{w} = \phi^{f} \left(\boldsymbol{v}^{f} - \boldsymbol{v}^{s}\right).   $$

### Finite element formulation

In the following equations, ***x***^*s*^, $\mathrm {d}\Omega _{0}^{s}$, *J*^*s*^, and ***F***^*s*^ are abbreviated to ***x***, d*Ω*_0_, *J*, and ***F***, respectively. The solid displacement ***u*** can be defined as

18 $$ \boldsymbol{u} = \boldsymbol{x} - \boldsymbol{X}^{s},  $$

and ***v***^*s*^ can be rewritten as

19 $$ \boldsymbol{v}^{s} = \dot{\boldsymbol{u}}.  $$

By considering the original solid configuration as the reference configuration of the mixture [9], the right Cauchy-Green tensor ***C*** and the Lagrangian strain ***E*** for the mixture can be defined as

20 $$\begin{array}{*{20}l} \boldsymbol{C} = \boldsymbol{F}^{T} \cdot \boldsymbol{F} \end{array} $$

21 $$\begin{array}{*{20}l} \boldsymbol{E} = \frac{1}{2}(\boldsymbol{C} - \boldsymbol{I}), \end{array} $$

where *T* indicates transposition. The first and second Piola-Kirchhoff stresses ***π*** and ***S*** are related to the total Cauchy stress ***σ*** by:

22 $$\begin{array}{*{20}l} \boldsymbol{\varPi} = J\boldsymbol{F}^{-1}\cdot \boldsymbol{\sigma} \end{array} $$

23 $$\begin{array}{*{20}l} \boldsymbol{S} = J \boldsymbol{F}^{-1} \cdot \boldsymbol{\sigma} \cdot \boldsymbol{F}^{-T} \end{array} $$

The second Piola-Kirchhoff stress ***S***^*E*^ can be defined for the stress induced by solid deformation using the same relation:

24 $$ \boldsymbol{S}^{E} = J \boldsymbol{F}^{-1} \cdot \boldsymbol{\sigma}^{E} \cdot \boldsymbol{F}^{-T}.  $$

Then,

25 $$ \boldsymbol{S} = -pJ \boldsymbol{C}^{-1} + \boldsymbol{S}^{E}.  $$

The boundary conditions were specified on a reference surface *Γ*_0_ for the mixture as a whole. The boundary conditions for the solid displacement and the surface traction are prescribed on $\Gamma _{0}^{u}$ and $\Gamma _{0}^{t}$, respectively. Here, $\Gamma _{0}^{u}$ and $\Gamma _{0}^{t}$ represent the complementary portions of *Γ*_0_:

26 $$\begin{array}{*{20}l} \Gamma_{0}=\Gamma_{0}^{u} \cup \Gamma_{0}^{t} \end{array} $$

27 $$\begin{array}{*{20}l} \Gamma_{0}^{u} \cap \Gamma_{0}^{t}=\emptyset. \end{array} $$

Likewise, the boundary conditions for the fluid pressure and the outward flow through the current surface are prescribed on $\Gamma _{t}^{p}$ and $\Gamma _{t}^{w}$, respectively. Here, $\Gamma _{t}^{p}$ and $\Gamma _{t}^{w}$ represent the complementary portions of the current whole surface *Γ*_*t*_:

28 $$\begin{array}{*{20}l} \Gamma_{t}=\Gamma_{t}^{p} \cup \Gamma_{t}^{w} \end{array} $$

29 $$\begin{array}{*{20}l} \Gamma_{t}^{p} \cap \Gamma_{t}^{w}=\emptyset. \end{array} $$

Generally, the solid and fluid boundary partitions do not need to coincide.

By using the Piola transformation (Appendix 1), Eq. (1) can be transformed into

30 $$ \boldsymbol{\nabla}_{\chi} \cdot \boldsymbol{\varPi} = \boldsymbol{0},   $$

where ***∇***_*χ*_ represents the gradient operator with respect to the reference configuration. An integral formulation can be obtained by multiplying Eq. (30) by an admissible displacement field $\boldsymbol {\check {u}}$ and integrating the result over a reference volume *Ω*_0_ of the mixture:

31 $$ \int_{\Omega_{0}} (\boldsymbol{\nabla}_{\chi} \cdot \boldsymbol{\varPi}) \cdot \boldsymbol{\check{u}} ~\mathrm{d}\Omega_{0} = 0.  $$

Another integral formulation is obtained using a different admissible displacement field $\boldsymbol {\check {u}}+\delta \boldsymbol {u}$. After some tensor manipulations and application of the Gauss theorem, the difference between these equations is replaced by

32 $$ \int_{\Omega_{0}} \boldsymbol{S} : \delta \boldsymbol{E} \mathrm{d}\Omega_{0} = \int_{\Gamma_{0}^{t}} \left(\boldsymbol{\varPi}^{T} \cdot \boldsymbol{N} \right) \cdot \delta \boldsymbol{u} \mathrm{d}\Gamma_{0}   $$

where *δ****E*** is defined consistently with *δ****u***, and ***N*** is the outward unit normal vector on the reference surface.

The integral formulation of Eq. (16) can be obtained by multiplying the equation and an admissible pressure field $\check {p}$ and integrating the result over a current volume *Ω*_*t*_:

33 $$ \int_{\Omega_{t}} \check{p} \boldsymbol{\nabla}_{x}\cdot\left(\dot{\boldsymbol{u}}+\boldsymbol{w} \right) \mathrm{d}\Omega_{t} = \int_{\Omega_{t}} \left[ \check{p} \boldsymbol{\nabla}_{x}\cdot\dot{\boldsymbol{u}} + \boldsymbol{\nabla}_{x}\cdot \left(\check{p}\boldsymbol{w}\right) - \boldsymbol{\nabla}_{x}\check{p}\cdot \boldsymbol{w} \right] \mathrm{d}\Omega_{t} = 0.  $$

By obtaining another integral formulation using a different admissible pressure field $\check {p}+\delta p$, and considering the difference between two equations,

34 $$ \int_{\Omega_{t}} \left[ \delta p \boldsymbol{\nabla}_{x}\cdot\dot{\boldsymbol{u}} - \boldsymbol{\nabla}_{x}\delta p\cdot \boldsymbol{w} \right] \mathrm{d}\Omega_{t} = -\int_{\Gamma_{t}^{w}} \delta p~ \boldsymbol{w} \cdot \boldsymbol{n} ~ \mathrm{d}\Gamma_{t}  $$

is obtained by using the Gauss theorem. Here, the term ***w***·***n*** in the right hand represents the outward flow through the surface per unit area. Since the surface of the mixture is covered by the membrane, the outward flow through the surface, that is, the transmembrane flow is determined by the permeability of the membrane, which is given as the boundary condition in this study. Assuming that the motion of a fluid inside the mixture follows Darcy’s law, ***w*** in the left hand can be obtained by

35 $$ \boldsymbol{w} = -\boldsymbol{\kappa}\cdot\boldsymbol{\nabla}_{x}~p,   $$

where ***κ*** is the Darcy permeability tensor of the mixture, and this equation can be replaced by

36 $$ \int_{\Omega_{t}} \left[ \delta p \boldsymbol{\nabla}_{x}\cdot\dot{\boldsymbol{u}} + \boldsymbol{\nabla}_{x}\delta p\cdot \boldsymbol{\kappa}\cdot\boldsymbol{\nabla}_{x}~p \right] \mathrm{d}\Omega_{t} = -\int_{\Gamma_{t}^{w}} \delta p~ \tilde q ~ \mathrm{d}\Gamma_{t}.   $$

where $\tilde q$ is the given outward flow.

### Linearizations

The set of Eqs. (32) and (36) can be discretized by finite element implementations. Within the *n*-th element obtained by subdividing the observed continuum into *n*_*el*_ elements, the displacement of the solid phase, ***u***, can be interpolated as follows:

37 $$ \boldsymbol{u} = \sum\limits_{i=1}^{n_{u}} N_{u}^{(i)}\boldsymbol{u}^{(i)},  $$

where *n*_*u*_ is the total number of nodes for ***u*** in the element, $N_{u}^{(i)}$ is the shape function of *i*-th node (see Appendix 2 for details), and ***u***^(*i*)^ is the *i*-th nodal displacement. In the same way, the fluid pressure, *p* can be interpolated as

38 $$ p = \sum\limits_{i=1}^{n_{p}} N_{p}^{(i)}p^{(i)},  $$

where *n*_*p*_ is the total number of nodes for *p* in the element, $N_{p}^{(i)}$ is the shape function of *i*-th node (see Appendix 2 for details), and *p*^(*i*)^ is the *i*-th nodal pressure. Their virtual values, *δ****u*** and *δp*, can be interpolated using $N_{u}^{(i)}$ and $N_{p}^{(i)}$, respectively:

39 $$ \delta \boldsymbol{u} = \sum\limits_{i=1}^{n_{u}} N_{u}^{(i)}\delta{\boldsymbol{u}}^{(i)}, \delta p = \sum\limits_{i=1}^{n_{p}} N_{p}^{(i)} \delta {p}^{(i)}.  $$

The solid displacement is isoparametric with the element coordinates:

40 $$ \boldsymbol{x} = \sum\limits_{i=1}^{n_{u}} N_{u}^{(i)}\boldsymbol{x}^{(i)}.  $$

By using the coefficients of ***u***^(*i*)^, ***E***, ***S***, and $\tilde {\boldsymbol {t}} (\equiv \boldsymbol {\varPi }^{T} \cdot \boldsymbol {N})$ at matrix notation, the following vectors can be defined at an arbitrary point:

41 $$\begin{array}{*{20}l} \{ u \} = \left\{ u_{1}^{(1)}~ u_{2}^{(1)}~ u_{3}^{(1)}~ u_{1}^{(2)}~ u_{2}^{(2)}~ u_{3}^{(2)}~ \dots~u_{1}^{(n_{u})}~ u_{2}^{(n_{u})}~ u_{3}^{(n_{u})}\right\}^{T} \end{array} $$

42 $$\begin{array}{*{20}l} \{ E \} = \{ E_{11}~E_{22}~E_{33}~2E_{12}~2E_{23}~2E_{31}\}^{T} \end{array} $$

43 $$\begin{array}{*{20}l} \{ S \} = \{ S_{11}~S_{22}~S_{33}~S_{12}~S_{23}~S_{31}\}^{T} \end{array} $$

44 $$\begin{array}{*{20}l} \{\tilde t\}=\left\{\tilde t_{1}~\tilde t_{2}~\tilde t_{3}\right\}^{T}. \end{array} $$

By defining the vectors {*δu*} and {*δE*} in the same way, matrix [*B*] can be obtained as a matrix that satisfies the following relationship:

45 $$ \{ \delta E \} = [\!B]\{ \delta u\}.  $$

Then, by defining the matrix [*N*_*u*_] as

46 $$ [N_{u}] =\left[ \begin{array}{cccccccccc} N_{u}^{(1)} & & &N_{u}^{(2)} & & & \cdots &N_{u}^{(n_{u})} & & \\ &N_{u}^{(1)} & & &N_{u}^{(2)} & & \cdots && N_{u}^{(n_{u})} & \\ & & N_{u}^{(1)} && & N_{u}^{(2)}& \cdots & & &N_{u}^{(n_{u})} \end{array}\right],  $$

Equation (32) can be rewritten as:

47 $$ \sum\limits_{n=1}^{n_{el}} \{ \delta u \}^{T} \int_{\Omega_{0}^{e}} [\!B]^{T}\{S\} \mathrm{d}\Omega_{0} = \sum\limits_{n=1}^{n_{el}} \{ \delta u \}^{T} \int_{\Gamma_{0}^{e}} [N_{u}]^{T} \{\tilde t\} ~\mathrm{d}\Gamma_{0},   $$

where $\Omega _{0}^{e}$ and $\Gamma _{0}^{e}$ denote the parts of *Ω*_0_ and $\Gamma _{0}^{t}$ included in the *n*-th element.

The vector of pressure nodal value can be defined as:

48 $$ \{ p \} = \left\{ p^{(1)}~ p^{(2)}~ \dots~ p^{(n_{p})}\right\}^{T},  $$

where vector {*δp*} is defined in the same way. The 1×*n*_*p*_ matrix [*N*_*p*_] can be defined as:

49 $$ [N_{p}] =\left[ \begin{array}{cccc} N_{p}^{(1)} &N_{p}^{(2)}& \cdots &N_{p}^{(n_{p})} \end{array}\right].  $$

By using the coefficients of ***x*** at matrix notation, the matrix [*z*] is defined as:

50 $$ [\!z] = \left[ \begin{array}{cccc} \frac{\partial{N}_{p}^{(1)}}{\partial{x}_{1}} &\frac{\partial{N}_{p}^{(2)}}{\partial{x}_{1}} & \cdots &\frac{\partial{N}_{p}^{(n_{p})}}{\partial{x}_{1}} \\ \frac{\partial{N}_{p}^{(1)}}{\partial{x}_{2}} &\frac{\partial{N}_{p}^{(2)}}{\partial{x}_{2}} & \cdots &\frac{\partial{N}_{p}^{(n_{p})}}{\partial{x}_{2}} \\ \frac{\partial{N}_{p}^{(1)}}{\partial{x}_{3}} &\frac{\partial{N}_{p}^{(2)}}{\partial{x}_{3}} & \cdots &\frac{\partial{N}_{p}^{(n_{p})}}{\partial{x}_{3}} \end{array}\right],  $$

Then, by defining [*κ*] as the coefficient matrix of *κ*, Eq. (36) leads to the following equation:

51 $$ \sum\limits_{n=1}^{n_{el}} \{ \delta p \}^{T} \int_{\Omega_{t}^{e}} \left([N_{p}]^{T} \boldsymbol{\nabla}_{x}\cdot\dot{\boldsymbol{u}} + [z]^{T}[\kappa][z] p \right)~\mathrm{d}\Omega_{t} = \sum\limits_{n=1}^{n_{el}} \{ \delta p \}^{T} \int_{\Gamma_{t}^{e}} [N_{p}]^{T} \tilde q ~\mathrm{d}\Gamma_{t}   $$

where $\Omega _{t}^{e}$ and $\Gamma _{t}^{e}$ denote the parts of *Ω*_*t*_ and $\Gamma _{t}^{w}$ included in the *n*-th element.

By defining $\{\delta \bar {u}\}$ and $\{\delta \bar p\}$ as vectors of all nodal values, Eqs. (47) and (51) can be rewritten as:

52 $$\begin{array}{*{20}l} \{\delta\bar{u}\}^{T} \{F_{\text{int}}\} = \{\delta\bar{u}\}^{T} \{F_{\text{ext}}\} \end{array} $$

53 $$\begin{array}{*{20}l} \{\delta \bar p\}^{T} \{\varLambda_{\text{int}}\} = \{\delta \bar p\}^{T} \{\varLambda_{\text{ext}}\} \end{array} $$

where the equivalent nodal vectors {*F*_ext_}, {*F*_int_}, {Λ_ext_}, and {Λ_int_} represent external force, internal force, external outward flow, and internal outward flow, respectively. Here, the external outward flow {Λ_ext_} is the value provided as the Neumann boundary condition representing the characteristics of the membrane which surrounded the mixture. For spatial integration, the Gauss integration can be used. As $\{\delta \bar u\}$ and $\{\delta \bar p\}$ are arbitrary vectors,

54 $$ \{F_{\text{int}}\} = \{F_{\text{ext}}\}, \hspace{20pt} \{\varLambda_{\text{int}}\} = \{\varLambda_{\text{ext}}\}.   $$

**Fig. 5:**
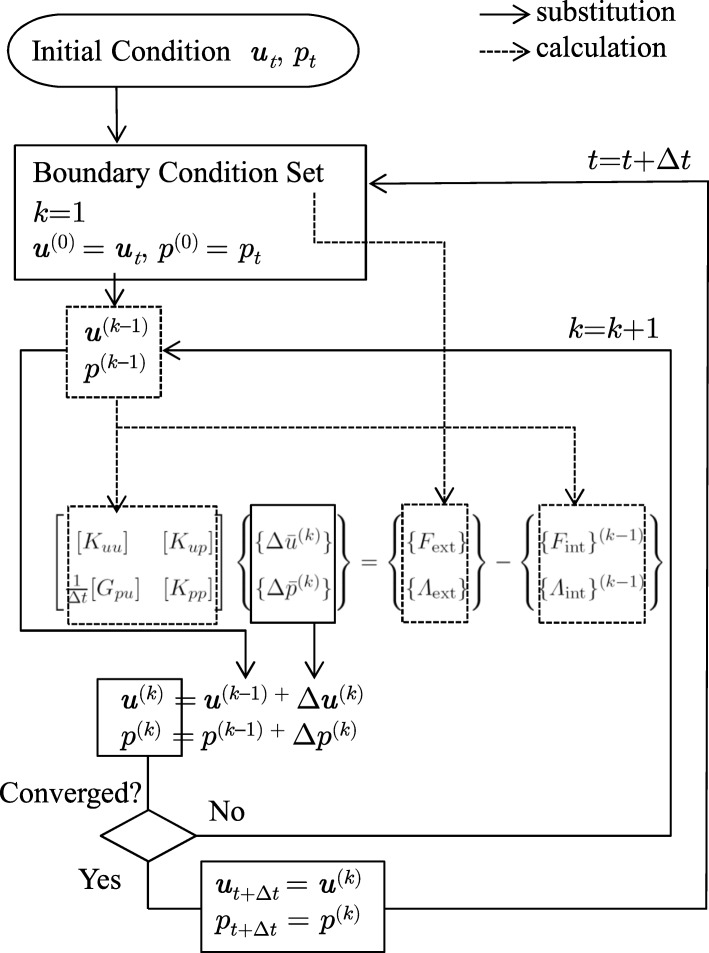
Algorithm of the iterative method. Solid arrows indicate that the value of the start point is assigned to the value of the end point. Dashed arrow indicates that the value of the start point is used to calculate the value of the end point

Since these systems of equations are generally nonlinear, we solved them by using an iterative method, Newton-Raphson, within each time increment *Δt*. By using the backwards Euler algorithm, $\dot {\boldsymbol {u}}$ at *t*+*Δt* can be replaced:

55 $$ \dot{\boldsymbol{u}}_{t+\Delta t} =\frac{\boldsymbol{u}_{t+\Delta t} - \boldsymbol{u}_{t}}{\Delta t},  $$

where the subscripts indicate time. The algorithm of the iterative method is presented in Fig. 5. When the solutions at previous time step *t* are known and represented as ***u***_*t*_ and *p*_*t*_, they are adopted as the initial predictors ***u***^(0)^ and *p*^(0)^ for the iterative scheme to obtain the solution at time *t*+*Δt*:

56 $$ \boldsymbol{u}^{(0)} = \boldsymbol{u}_{t}, \hspace{20pt} p^{(0)} = p_{t}.  $$

The *k*-th predictors ***u***^(*k*)^ and *p*^(*k*)^ are

57 $$ \boldsymbol{u}^{(k)} = \boldsymbol{u}^{(k-1)}+ \Delta{\boldsymbol{u}}^{(k)}, \hspace{20pt} p^{(k)} = p^{(k-1)}+ \Delta p^{(k)}  $$

where *Δ****u***^(*k*)^ and *Δp*^(*k*)^ are the *k*-th incremental values. By arranging *Δ****u***^(*k*)^ and *Δp*^(*k*)^ at all nodes in a row as the vectors $\{\Delta \bar {u}\}^{(k)}$ and $\{\Delta \bar {p}\}^{(k)}$, respectively Eq. (54) can be linearized as:

58 $$\begin{array}{*{20}l} [K_{uu}] \{\Delta\bar{u}\}^{(k)} + [K_{up}] \{\Delta\bar{p}\}^{(k)} = \{F_{\text{ext}}\}- \{F_{\text{int}}\}^{(k-1)} \end{array} $$

59 $$\begin{array}{*{20}l} \frac{1}{\Delta t}[G_{pu}] \{\Delta\bar{u}\}^{(k)} + [K_{pp}] \{\Delta\bar{p}\}^{(k)} = \{\varLambda_{\text{ext}}\}- \{\varLambda_{\text{int}}\}^{(k-1)}, \end{array} $$

where the matrices in the left hand represent the stiffness matrices. The vectors {*F*_int_}^(*k*−1)^ and {Λ_int_}^(*k*−1)^ are {*F*_int_} and {Λ_int_}, respectively, calculated using the predictors obtained at the previous iteration, ***u***^(*k*−1)^ and *p*^(*k*−1)^. For the *k*-th iteration, the system of equations that is obtained by coupling these two systems of equations is solved for $\{\Delta \bar {u}\}^{(k)}$ and $\{\Delta \bar {p}\}^{(k)}$:

60 $$ \left[\begin{array}{cc} [K_{uu}] & [K_{up}] \\ \frac{1}{\Delta t}[G_{pu}] & [K_{pp}] \\ \end{array}\right] \left\{\begin{array}{cc} \{ \Delta\bar{u}^{(k)} \} \\ \{ \Delta \bar p^{(k)} \} \end{array}\right\} = \left\{\begin{array}{cc} \{ F_{\text{ext}}\}\\ \{ \varLambda_{\text{ext}} \} \end{array}\right\} - \left\{\begin{array}{cc} \{F_{\text{int}}\}^{(k-1)} \\ \{ \varLambda_{\text{int}}\}^{(k-1)} \end{array}\right\}   $$

The iteration continues until a convergence criterion is satisfied, and the converged solution is assumed as the solution at the time step *t*+*Δt*. For this present analysis, the following convergence criterion of the norm of the incremental vector was used:

61 $$ \left|\begin{array}{c} \Delta\bar{u}^{(k)} \\ \Delta \bar p^{(k)} \end{array}\right| ~<~ TOL \left|\begin{array}{c} \Delta\bar{u}^{(1)} \\ \Delta \bar p^{(1)} \end{array}\right|  $$

where *TOL* is a user-specified tolerance with the value of 10^−5^ in this study.

## Results

We conducted stress relaxation analysis using the described analysis method. For the drained solid constituent, we chose a linear isotropic constitutive model and assumed homogeneous samples, although a more realistic model can be employed. We assumed that the drained solid skeleton is a hyperelastic material, which has the elastic potential function *W*:

62 $$ S^{E}_{ij}=\frac{\partial{W}}{\partial{E}_{ij}},  $$

where the coefficients of ***S***^*E*^ and ***E*** at matrix notation were used. We employed the following hyperelastic energy function:

63 $$ W = \frac{\lambda}{2} (\text{tr} \boldsymbol{E})^{2} + \mu\,\text{tr}(\boldsymbol{E}^{2})   $$

where *λ* and *μ* represent Lamé’s constants. Here, ***S***^*E*^ is linear to ***E***:

64 $$ \boldsymbol{S}^{E} = \lambda (\text{tr} \boldsymbol{E}) \boldsymbol{I} + 2\mu\boldsymbol{E}.  $$

The hyperelastic properties dominate the solid displacements, fluid velocities, and the pore water pressures throughout the deformation process. Although the dense solid constituting the skeleton is incompressible, the skeleton is compressible because it is porous and contains compressible voids. Thus, we assumed that Poisson’s ratio of the skeleton is 0.0. We also assumed that the skeleton is homogeneous with Young’s modulus = 0.3 kPa (i.e., *λ*=0.0 and *μ*=0.15 kPa), an initial solid volume fraction $\phi ^{s}_{0}=$0.2, and a permeability ***κ***=*κ****I*** (*κ*=8.0 m^4^/N-s). As indicated in Eqs. (35) and (36), the permeability ***κ*** affects the fluid and solid velocities, ***w*** and $\dot {\boldsymbol {u}}$, resulting in a viscous behavior. With smaller ***κ***, the fluid velocity inside the mixture becomes lower. Except specific conditions, the lower fluid velocity makes solid velocity lower. In general, this kind of viscous behavior may absorb a shock of impact by reducing accelerations of objects. The viscous behavior can protect the brain from the hit of skull in the SAS when considering the trabecular network filled with CSF as the hydrated soft tissue.

**Fig. 6:**
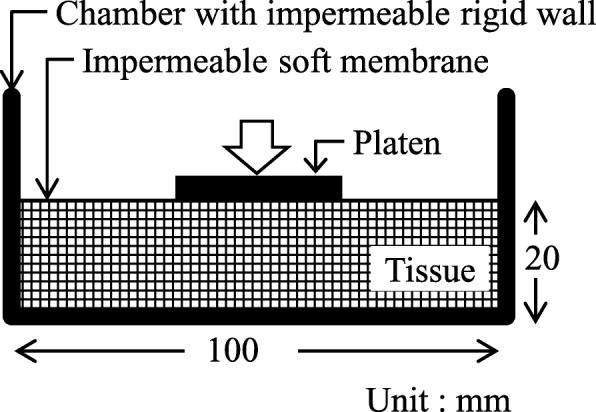
Confined compression test. The tissue of 100 mm × 100 mm × 20 mm size, which is surrounded by a flaccid impermeable membrane, is in a rigid chamber. The tissue is compressed by a platen covering the central part of the top surface

**Fig. 7:**
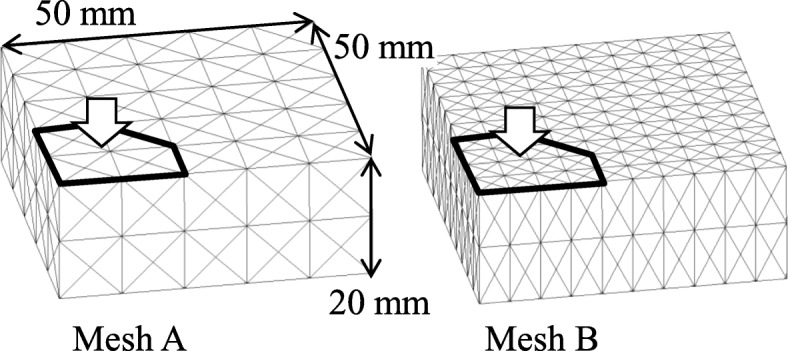
Finite element meshes. The axisymmetric finite element mesh, which represents 1/4 of the tissue, consists of 1200 (Mesh A) or 4800 (Mesh B) elements. The platen covered the areas surrounded by bold lines, which may bulge with the mesh during deformation

One of the simplest tests to study the behavior of the hydrated soft tissues is the one-dimensional confined compression test [7–9], where a tissue is surrounded by a rigid and impermeable chamber, and the permeable platen is loaded at the top. However, the aim of the present study was to develop a method for the analysis of tissues surrounded by deformable membranes that control transmembrane flows. Therefore, we covered the top surface of the tissue by the flaccid impermeable membrane, i.e., set to {Λ_ext_}=***0***, which has not ever been represented using the FEMs formulated by Suh et al. [8] and Levenston et al. [9] by the reason mentioned previously (Fig. 6). The permeability of the membrane, which is given as the boundary condition in this study, changes the total volume of the mixture by determining the transmembrane flow. It may result in an edema. Although the membrane is assumed to be flaccid, it cannot deform when the top surface is uniformly loaded because the tissue cannot deform due to its incompressibility. Thus, we conducted a three-dimensional test, where the platen covered only the central part of the top surface. The platen was compressed at the velocity of 6 m/s during 1 ms of the compression period, and maintained at that compression level, 6 mm, during the following hold period. The finite element meshes used in the analyses represent 1/4 of the tissue (Fig. 7). The platen axially pushed the areas surrounded by bold lines. At the other area of the top surface, the load perpendicular to the surface was kept at 0, i.e., ***π***^*T*^·***N***=***0***. The displacements perpendicular to the surface were confined at the sides and base. We prepared two types of meshes, Mesh A and Mesh B, which consisted of 1200 and 4800 5/4c displacement-pressure elements, respectively. Three different sets of time steps and mesh types used for the numerical examples are presented in Table 1.

**Table 1:** Time stepts and mesh types used for the numerical examples

Parameter	Case 1	Case 2	Case 3
*Δ* *t*	0.1 ms	0.2 ms	0.2 ms
Mesh type	Mesh A	Mesh A	Mesh B

**Fig. 8:**
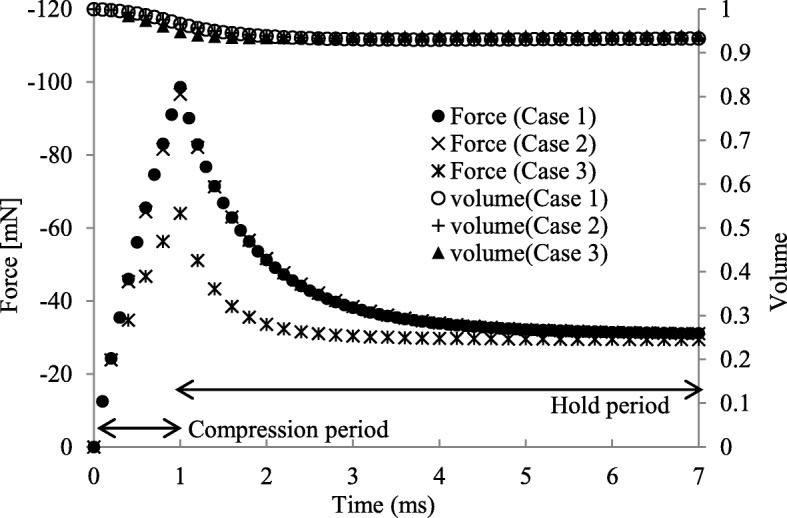
Time histories of the platen reaction force and the total volume of the analyzed tissue. The total volumes are normalized using the original volumes

Time histories of the reaction forces on the platen and the total volumes of the analyzed tissues are shown in Fig. 8. The results obtained in cases where Mesh A was used, i.e., Case 1 and Case 2, were shown to be consistent. Additionally, these results were in quite good agreement with those obtained in Case 3 using Mesh B, in terms of the total volume history and converged reaction force. However, the peak value of the reaction force in Case 3 was 66% of those obtained in Case 1 and Case 2. Although this inconsistency suggests the necessity of the finer mesh, which requires a huge amount of calculation or an effective matrix solver, we employed the direct matrix solver and did not explore this issue in the present study. We treated the mesh refinement and the quantitative discussion as future tasks and discussed just the qualitative behaviors observed in all of the three analyses.

**Fig. 9:**
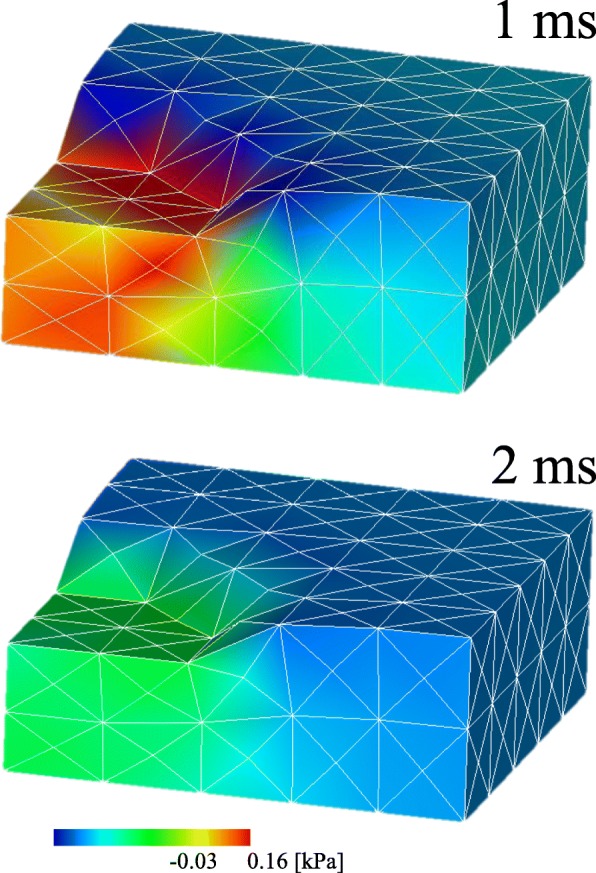
The color contour of the pore water pressure at 1 ms and 2 ms in Case 2

**Fig. 10:**
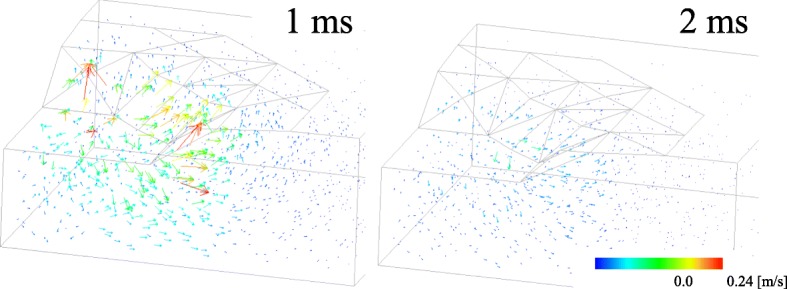
The distributions of the pore water flow velocities at 1 ms and 2 ms in Case 2. Arrow colors indicate flow speeds

**Fig. 11:**
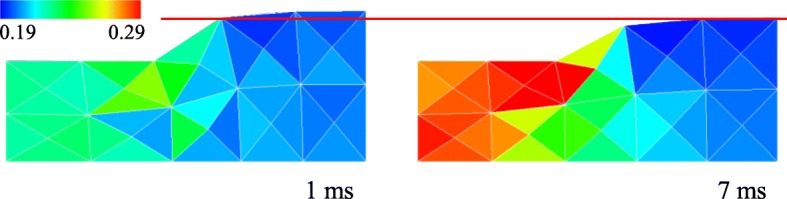
Vertical sectional view of the tissues at 1 ms and 7 ms in Case 2. The colors indicate the solid volume fraction of the element. Red line indicates the initial height of the tissue

In Case 2, according to the pore pressure gradient caused by the compression during the compression period (Fig. 9), fluid moved out from the compressed area to the surrounding areas (Fig. 10). As shown in Fig. 11, the solid volume fraction *ϕ*^*s*^ increased in the area under the platen due to the outflow of fluid, and decreased under the surface around the platen because of the fluid block and accumulation by the impermeable membrane. The accumulated fluid led to the swelling of the tissue around the platen at the end of the compression period (1 ms). During the hold period, the movement of the fluid relaxed the pore water pressure gradient (Fig. 9), which resulted in the reduction of the reaction force (Fig. 8) and the fluid velocity (Fig. 10). Afterwards, the reaction force converged to the value derived from the elastic deformation of the solid phase, showing a typical stress relaxation (Fig. 8) until the fluid stopped. The similar behaviors were observed in other two analyses.

The total volume decreased to 93.3% at the end of the compression period (Fig. 8), against the expectation that the total volume is maintained due to the impermeable membrane covering the entire tissue. Additionally, the surface around the platen sank during the hold period, while the total volume remained stable and no counter flow occurred, i.e., the fluid flow to compressed area from the surrounding areas.

## Discussion

Here, based on the mixture theory, we performed the finite element formulation of a hyperelastic porous solid filled with fluid, which flowed according to the pore water pressure gradient and interacted with the deformation of the solid. In the method developed and presented here, only the solid displacements and the pore water pressures are treated as the nodal unknowns and the fluid velocities are not regarded as such. Therefore, the fluid flow volume across the membrane can be directly given as the Neumann boundary condition. Our new method enables us to analyze a large deformation of hydrated tissue surrounded by a deformable membrane that controls transmembrane flow. While Oomens et al. [7], who also used the set of the solid displacements and the pore water pressures for the nodal unknowns, employed a rate-type constitutive equation for the solid phase, we assumed that the drained solid skeleton is a hyperelastic material that has elastic potential function.

Using our new method, we analyzed the hydrated soft tissues surrounded by flaccid impermeable membranes. Our new method has the following three limitations based on the simulation results. Firstly, we could neither validate our proposed method using a simple problem with an analytical solution, such as one-dimensional compression, nor compare our results with the results obtained by previously presented numerical methods since there are no studies on numerical methods of the hydrated biological soft tissues surrounded by deformable membranes that control the transmembrane flows. Further studies are needed for validation of our proposed study using some sort of analytical solutions or experimental data. Secondly, although the whole tissue was surrounded by the fully impermeable membrane, the total volume decreased to 93.3% by the end of the compression period, showing that the results contain numerical errors. The errors should be investigated and corrected in the future. Thirdly, the peak values of the reaction force were shown to depend on the mesh. This is probably because too coarse mesh failed to capture the nonlinear pressure gradient around the membrane, generating the inconsistency of the reaction force. Further investigation is necessary for the problem of mesh dependency.

Despite above mentioned limitations of our new method, the fluid extruded by platen was trapped by the impermeable membrane and it led to the swelling of the tissue around the platen during the compression period, while the total volume was well maintained during the hold period, suggesting the ability of our method to reproduce a deformable membrane that controls transmembrane flow.

Fluid flowed according to the pore water pressure gradient caused by the compression, resulting in the relaxation of the gradient. Afterwards, the reaction force and the fluid velocity were reduced and converged to the constant values. As a result, the reaction force showed a typical stress relaxation although the constitutive equation of the solid skeleton did not contain the viscoelastic component and the permeability was constant. This viscoelastic behavior can play important roles in the body. For example, the viscoelasticity of SAS can protect the brain by absorbing shocks. As the behavior involves the volume changes of elements with the total volume preserved by the interactions between elements via the movement of the fluid, it is impossible to reproduce this viscoelastic behavior by substituting an equivalent viscoelastic body for the mixture material used in this study.

## Conclusions

To analyze the hydrated soft tissues surrounded by deformable membranes that control transmembrane flows, which are observed in most of the tissues, we developed a new nonlinear FEM based on mixture theory using the pore water pressure and solid displacement as the nodal unknowns, and controlled the transmembrane flows across a deformable membrane with the Neumann boundary condition. The results obtained by our new method demonstrated that our new method can effectively analyze large deformation of hydrated tissues surrounded by a flaccid impermeable membrane. Although the proposed method has some limitations on the validation, numerical errors, and mesh dependency, it is confirmed that the proposed method can reproduce the viscoelastic behavior characterized in hydrated biological soft tissues that the fluid trapped by the impermeable membrane led to the swelling of the tissue around the platen. Further developments of this method may allow the analysis of the biological phenomena such as brain edema, brain trauma, the flow of blood and lymph in capillaries and pitting edema.

## Appendix 1

### Piola transformation

The Piola transformation is a fundamental operation relating two descriptions in continuum mechanics, and can be performed on any index of tensors [21]. Let ***A*** be a tensor defined by

65 $$ \boldsymbol{A}=J\boldsymbol{F}^{-1}\cdot \boldsymbol{a}  $$

where ***A*** is an arbitrary tensor. When ***n*** represents the outward unit normal vectors of the micro surface d *Γ*_*t*_ on the current configuration, the Nanson’s formula can be denoted as:

66 $$ \boldsymbol{n} \mathrm{d}\Gamma_{t} = J \boldsymbol{F}^{-T}\cdot \boldsymbol{N} \mathrm{d}\Gamma_{0},  $$

where ***N*** represents the outward unit normal vectors of the micro surface d *Γ*_0_ on the reference configuration. Using these equations, the following relationship is obtained:

67 $$ \boldsymbol{a}^{T} \cdot \boldsymbol{n} \mathrm{d}\Gamma_{t} = J \boldsymbol{a}^{T} \cdot \boldsymbol{F}^{-T}\cdot \boldsymbol{N} \mathrm{d}\Gamma_{0} = \boldsymbol{A}^{T} \cdot \boldsymbol{N} \mathrm{d}\Gamma_{0}.   $$

By using the Gauss theorem on the reference configuration, Eq. (67), the Gauss theorem on the current configuration, and Eq. (3) in turn, the following relationship can be obtained:

68 $$ \int_{\Omega_{0}} \nabla_{\chi} \cdot \boldsymbol{A} \mathrm{d}\Omega_{0}= \int_{\Gamma_{0}} \boldsymbol{A}^{T} \cdot \boldsymbol{N} \mathrm{d}\Gamma_{0} = \int_{\Gamma_{t}} \boldsymbol{a}^{T} \cdot \boldsymbol{n} \mathrm{d}\Gamma_{t} = \int_{\Omega_{t}} \boldsymbol{\nabla}_{x} \cdot \boldsymbol{a} \mathrm{d}\Omega_{t} = \int_{\Omega_{0}} J\boldsymbol{\nabla}_{x} \cdot \boldsymbol{a} \mathrm{d}\Omega_{0}.  $$

As this equation holds for any arbitrary parts, we obtained the relationship

69 $$ \nabla_{\chi} \cdot \boldsymbol{A} = J\boldsymbol{\nabla}_{x} \cdot \boldsymbol{a}.  $$

## Appendix 2

### Shape functions in a 5/4c displacement-pressure element

As the continuity Eq. (36) involved the second derivative of the pressure field, *C*^0^ continuity was required for the pressure field. In addition, the mixed formulation requires a suitable combination of the displacement and pressure interpolations in order to obtain a robust element. In the present analysis, 5/4c (*P*1 + /*P*1) displacement-pressure element, which is in the family of MINI elements, is used. This is a tetrahedral first-order element with a linear continuous interpolation. The pressure can be interpolated by 4 nodes located at vertices. For the interpolation of the displacement, a bubble node located at the centroid was added as the 5th node.

With the surfaces of the original tetrahedron, a point P in the tetrahedron forms 4 subtetrahedrons. By defining the volume ratios of the subtetrahedrons to the original tetrahedron as *r*_*i*_,(*i*=1 to 4), the volume coordinate of the P can be defined as (*r*_1_,*r*_2_,*r*_3_). By using the volume coordinate, the shape functions of the displacement and the pressure are defined as

70 $$\begin{array}{@{}rcl@{}} N_{u}^{(1)} &=& r_{1} - 64 r_{1} r_{2} r_{3} (1-r_{1} -r_{2} - r_{3})  \\ N_{u}^{(2)} &=& r_{2} - 64 r_{1} r_{2} r_{3} (1-r_{1} -r_{2} - r_{3})  \\ N_{u}^{(3)} &=& r_{3} - 64 r_{1} r_{2} r_{3} (1-r_{1} -r_{2} - r_{3})  \\ N_{u}^{(4)} &=& (1-r_{1} -r_{2} - r_{3}) - 64 r_{1} r_{2} r_{3} (1-r_{1} -r_{2} - r_{3})  \\ N_{u}^{(5)} &=& 256 r_{1} r_{2} r_{3} (1-r_{1} -r_{2} - r_{3}) \end{array} $$

71 $$\begin{array}{@{}rcl@{}} N_{p}^{(1)} &=& r_{1}  \\ N_{p}^{(2)} &=& r_{2}  \\ N_{p}^{(3)} &=& r_{3}  \\ N_{p}^{(4)} &=& 1-r_{1} -r_{2} - r_{3}. \end{array} $$
